# The Real Life of Ataxia Patients Without a Vertical Family History: a Twenty-year Experience in South Brazil

**DOI:** 10.1007/s12311-026-02067-2

**Published:** 2026-08-19

**Authors:** Carlos Alberto Moura Aschoff, Thiago Oliveira Silva, Ali Hasan, Elaine Migliorini, Karina Carvalho Donis, Rare Genomes Project Consortium, David Pellerin, Maria Luiza Saraiva-Pereira, Sandra Leistner, Fabiano Poswar, Roberto Giugliani, Cristina Brinckmann Oliveira Netto, Patrícia Ashton-Prolla, Jonas Alex Morales Saute, Laura Bannach Jardim

**Affiliations:** 1 Programa de Pós-Graduação em Genética e Biologia Molecular, Universidade Federal do Rio Grande do Sul, Avenida Bento Gonçalves 9500, Porto Alegre, 91501-970 Brazil; 2 Centro de Pesquisa Clínica do Hospital de Clínicas de Porto Alegre, Rua Ramiro Barcelos 2350, Porto Alegre, 90035-007 Brazil; 3 Serviço de Genética Médica do Hospital de Clínicas de Porto Alegre, Rua Ramiro Barcelos 2350, Porto Alegre, 90035-007 Brazil; 4 Programa de Pós-Graduação em Ciëncias Médicas, Universidade Federal do Rio Grande do Sul, Porto Alegre, Brazil; 5 Departamento de Genética, Universidade Federal do Rio Grande do Sul, Avenida Bento Gonçalves 9500, Porto Alegre, 91501-970 Brazil; 6 Hospital Israelita Albert Einstein, Avenida Albert Einstein 627, São Paulo, 05652-900 Brazil; 7 Department of Neurology and Neurosurgery, Montreal Neurological Hospital and Institute, McGill University, 3801 University Street, Montreal, QC H3A 2B4 Canada; 8 Dasa Genomica, Avenida Divino Salvador 876, São Paulo, 04089-001 Brazil; 9 Casa dos Raros, Rua São Manoel 730, Porto Alegre, 90610-261 Brazil; 10 Instituto Nacional de Ciência e Tecnologia em Prevenção e Saúde de Precisão em Oncogenética (INCT Prev-Onco), Rua Ramiro Barcelos 2350, Porto Alegre, 90035-007 Brazil; 11 Departamento de Medicina Interna, Universidade Federal do Rio Grande do Sul, Rua Ramiro Barcelos 2400, Porto Alegre, 90035-003 Brazil

**Keywords:** Hereditary ataxias, Recessive ataxias, Ataxia simplex, Healthcare delay, Healthcare access, Next generation sequencing

## Abstract

Studies of hereditary ataxias (HA) without vertical family history are necessary for designing diagnostic protocols. We described the 20 years’ experience of a Brazilian reference service with these cases through a retrospective cohort study of subjects evaluated from 2002 to 2020 in a university hospital. Tests for Friedreich ataxia, alpha-fetoprotein, common dominant ataxias, and brain imaging were the first steps, whereas ataxia Sanger panel, exome or genome sequencings (NGS) were the last ones. The outcomes were: diagnosis; no diagnosis after NGS; or incomplete diagnostic investigation. Diagnoses and diagnostic yields were also presented. 174 subjects started investigation and 120 came to the second visit: 45/120 received a diagnosis, 12/120 finished their investigation without a diagnosis, and 63/120 were incompletely investigated. Higher-than-expected proportions of white subjects and of people coming from small communities were found. Most common diagnoses were Friedreich ataxia, ataxia-telangiectasia, Coenzyme Q10 deficiency, Niemann-Pick type C, ataxia with oculoapraxia type 2, and spinocerebellar ataxia type 2. Thirty-two subjects were investigated by NGS; among them, 5/7 ataxia Sanger panels, 9/17 exome and 1/7 genome sequencings got a molecular diagnosis, with diagnostic yields of 71.4%, 52.9% and 14%, respectively. The high proportion of patients lost to follow-up, cases with incomplete investigation and white individuals suggest problems in the access to healthcare. Exome and Sanger panels were the most efficient methods for reaching a diagnosis. The fact they are not easily available in the public health system is an important barrier to overcome, hopefully soon.

**Supplementary Information:**

The online version contains supplementary material available at https://doi.org/10.1007/s12311-026-02067-2.

## Introduction

Hereditary ataxias (HA) are a large group of diseases whose common denominator is the presence of motor incoordination and monogenic inheritance. With highly heterogeneous etiologies, HA diagnosis usually requires an elaborate and extensive investigation plan [1–4]. Cases with autosomal dominant inheritance, generally with onset in adulthood, are more easily identified and called spinocerebellar ataxias (SCAs) [4, 5]. Investigation protocols for SCAs are already established, beginning with those due to expanded repetitive nucleotide sequences. A small proportion of SCA cases have no prior family history, either due to loss of information or *de novo* expansions [5–7].

Detecting recessive and X-linked inheritance can be more elusive. The small size of current urban sibships hinders horizontal recurrence, a hallmark of recessive diseases. Fragile X-associated tremor/ataxia syndrome (FXTAS), the most relevant X-linked form, occurs in people over 60 who have an *FMR1* premutation, most frequently without a previous family history [8]. In summary, isolated cases without a family history, so-called simplex cases, may be due to autosomal dominant, autosomal recessive or X-linked forms of ataxia.

There is still no clear consensus on the best diagnostic protocol for autosomal recessive and X-linked ataxias [1, 9, 10]. Complex diagnostic algorithms have been built in the last years and varied due to technological and knowledge advances, but also to differences in decision criteria— clinical characteristics, general blood tests, MRI, family history, cerebrospinal fluid and/or histological aspects, combined in different sequences [11–13]. A useful approach to start is to base the investigation on the age of onset of incoordination and to take advantage of the available epidemiological information [11–14]. In Latin America, classical Ataxia-telangiectasia (A-T) and Friedreich's ataxia (FRDA) appeared to be the most common forms of ataxia with onset in early childhood and adolescence-young adulthood, respectively [15]; the most common SCAs were SCA3/MJD, SCA2, SCA7 and SCA10 [16, 17]. Another attempt to simplify the problem was the construct "primary hereditary ataxia", intended to exclude hereditary multisystem disorders in which ataxia may be observed but is usually not the primary presenting manifestation [4]. This construct, however, does not solve the real-life problem, where doctors cannot always discern between primary and secondary ataxias for a patient that needs diagnosis. Quite a few HA—such as classical A-T [18], Dandy-Walker [19] and Joubert syndromes [20]—have specific clinical, biochemical and neuroimaging findings sufficient to establish a strong suspicion or diagnosis. The remaining are the vast majority and need molecular confirmation. The advent of next-generation sequencing (NGS), comprising targeted gene panels, exome and genome sequencing, came to simplify and increase efficiency of investigation. However, this remains out of reach, as these tests are still inaccessible to the public health system, at least in Brazil.

With a minimum of inclusion criteria, this study aimed to describe the experience of our referral center on hereditary ataxias without vertical inheritance since 2002. We reported the number of subjects who received a diagnosis, the number who did not receive a diagnosis despite having undergone complete investigation (with NGS), and the number of subjects with pending (incomplete) investigation in 2024, the end of the observation period.

## Methods

This was a retrospective review of the medical records of cases without a vertical family history, who developed ataxia between years 2002 and 2020 and sought genetic diagnosis at our institution between 2003 and 2021. Our center is the Reference Service for Rare Diseases in the state of Rio Grande do Sul for the Brazilian public health system, called Sistema Único de Saúde (SUS). It is located in Hospital de Clínicas de Porto Alegre and has a regional coverage for 11 million inhabitants. The review of medical records was approved by the local ethics committee (CAAE53405421.6.0000.5327). No contact was made with the subjects, and permission to waive the requirement for informed consent was obtained from the local Institutional Review Board.

Cases were retrieved from three databases securely stored at our institution: (1) subjects who underwent molecular study to diagnose FRDA—i.e., GAA repeat measurements in *FXN*; (2) ataxic children investigated in the oncogenetics outpatient clinics, as classical A-T families were usually followed up there due to their risk of having cancer; and (3) subjects investigated in the neurogenetics outpatient clinics. Inclusion criteria were presence of gait ataxia on neurological examination; absence of vertical recurrence in the family; and absence of acquired neurological disease.

The patients treated during the study period—2003 to 2024—were followed by different teams who did not use a unique structured diagnostic protocol, since throughout the period several laboratory tests were incorporated. To standardize the cases analysed by this study, subjects should have performed at least one of three studies in the first level of their investigation: GAA repeat measurements in *FXN*; serum concentration of alpha-fetoprotein (AFP), in cases with onset soon after the patient started to sit and walk, and before the 4 years of age, to screen for classic A-T and some forms of ataxia with ocular apraxia (AOA); and the local panel for SCAs—SCA1, SCA2, SCA3/MJD, SCA6, SCA7, and SCA10, in subjects with ataxia onset after the 18 years of age. These laboratory studies were done in-house; patients were entitled to have these tests free of charge, as they were covered by SUS. Subjects who came to the first visit but did not undergo either of them were considered primary losses. To establish a diagnosis of classical A-T, the combination of progressive cerebellar ataxia starting in childhood, oculomotor apraxia, telangiectasias, high levels of AFP, reduced immunoglobulins and frequent sinopulmonary infections was considered sufficient. The detection of specific biochemical or neuroimaging findings were considered sufficient to diagnose an IEM or a malformative condition, respectively. To all other causes of HA, the diagnostic criteria was the identification of the causal mutation. A minority of Sanger panels targeted to spastic paraplegias (SPG) or to Niemann-Pick type C (NPC) were done in-house during time-limited research studies. Sanger panels targeted on ataxias, exome and genome sequencing —hereinafter collectively referred to as NGS studies—were considered the most definitive diagnostic tools with the highest diagnostic yield; these were performed outside our institution and the public health system, whether through private payment, as complimentary tests, or via court order.

**Fig. 1:**
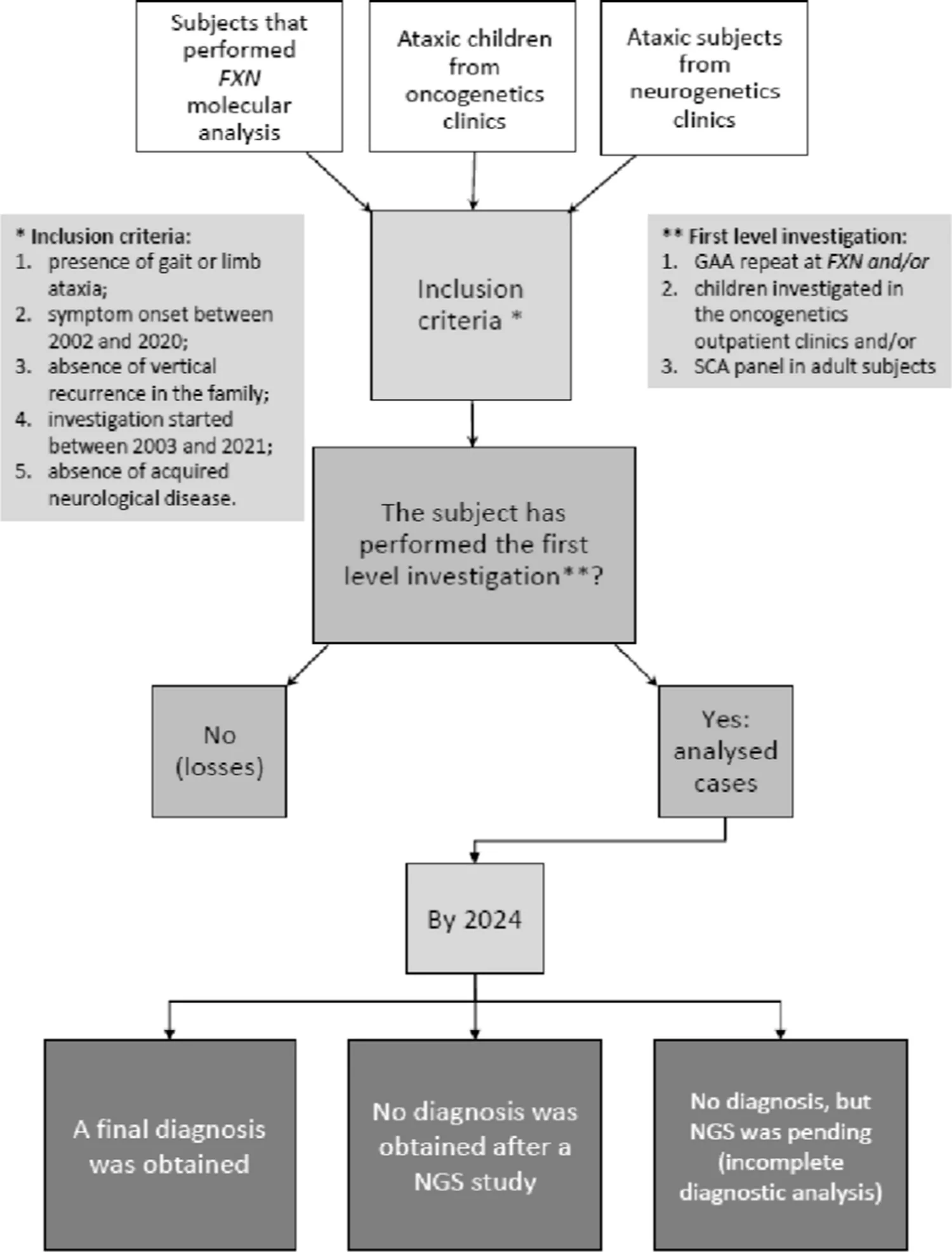
Algorithm used to define the outcomes of the present study

At the end of the study, in 2024, three general outcomes were possible for each individual: a final diagnosis; no diagnosis after a complete investigation that included some NGS study; or no diagnosis with pending (incomplete) investigation, for those subjects still waiting for a NGS study. Figure 1 describes the algorithm used to define the outcomes of the present observation.

Several independent variables were sought from the medical records that could impact on the success of the outcome: age, sex, year of birth, age at onset of symptoms (AOfs), year of onset of symptoms, recurrence among siblings, consanguinity between parents, place of residence; age and year at the start of the investigation, age and year at definitive diagnosis or last consultation, neurological examination, complementary blood tests, peripheral neurophysiology, and neuroimaging (Supplemental Material 1).

As secondary outcomes, the diagnostic delays since the AOfs and since the beginning of the investigation, a description of the diagnoses and their clinical characteristics, and the diagnostic yields of the complementary tests were also reported.

Descriptive data were presented as medians (range); exceptions were described in the text. Qualitative variables were compared using the chi-square test; quantitative variables were examined using Mann–Whitney U test and Kruskal–Wallis test with Dunn due to their non-parametric distributions. Statistical analyses were performed using PASW statistics (SPSS) version 2018, with a significance level of 0.05.

## Results

This retrospective review identified 174 subjects with gait ataxia starting between 2002 and 2020, without vertical recurrence in the family or acquired neurological diseases, who came for a first consultation between 2003 and 2021. One hundred-twenty subjects performed the first level investigation and were included, while the other 54 did not perform it and were considered primary losses (Supplemental Material 2).

**Fig. 2:**
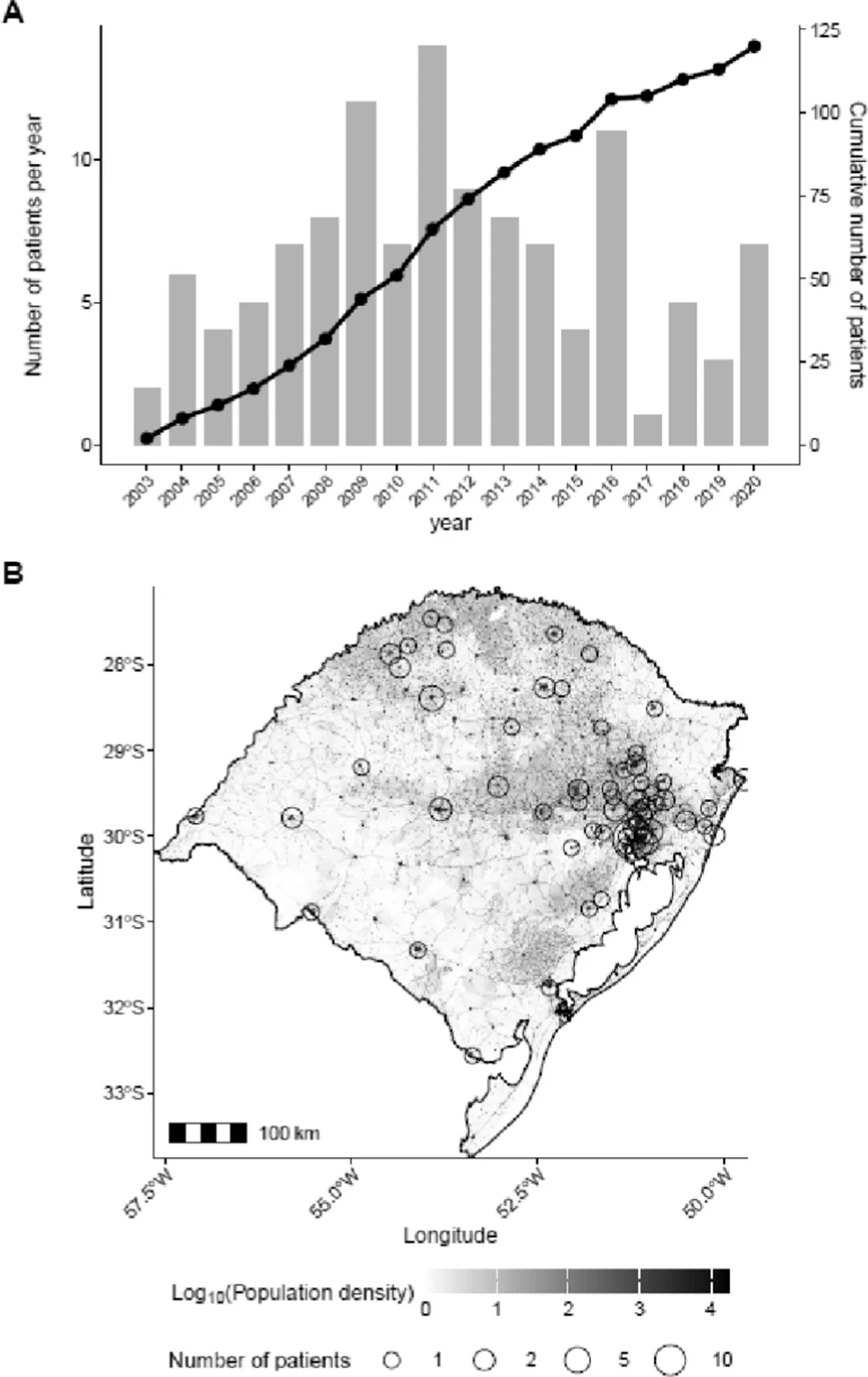
Spatiotemporal distribution of the subjects studied in this review. (**A**) Number of subjects per year at the start of their evaluations. (**B**) Demographic map of Rio Grande do Sul state with the place of residence of 120 ataxic subjects included in this study

The general characteristics of the 120 subjects that performed the first level of investigation, from now on referred to as cases “studied”, are presented in Table 1. Sixty-nine (57.5%) were female, a proportion similar to the expected for the general population (ns, chi-square). Figure 2 depicts the number of subjects per year at the onset of their evaluations and their places of residence in Rio Grande do Sul. Table 2 shows the proportion of the self-declared ethnicity and of the residence origins of both the overall 174 ataxic subjects that came to the first visit and of the 120 studied cases that performed at least the first level of investigation, compared with the general population of Rio Grande do Sul.

**Table 1:** General characteristics of the subjects that performed the first level of ataxia investigations and were included in the analyses

Age at onset of symptoms, in years *Median (range)* *n*		18.5 (0 to 67) 118
Age at onset of symptoms *n (%)*	0 to 11 years	42/118 (35%)
	12 to 20	23/118 (19.2%)
	21 to 40	23/118 (19.2%)
	More than 40	30/118 (25%)
Age at the start of evaluation, in years *Median (range)* *n*		23 (2 to 76) 120
Age at the start of evaluation *n (%)*	0 to 11 years	34/120 (28.3%)
	12 to 20	20/120 (16.7%)
	21 to 40	26/120 (21.7%)
	More than 40	40/120 (33.3%)
Consanguineous parents *n (%)*		19/109 (15%)
Recurrence in their sibship *n (%)*		29/109 (24.2%)
Outcome *n (%)*	Finished the evaluations without a diagnosis	12/120 (10%)
	Finished the evaluations with a diagnosis	45/120 (37.5%)
	Did not finish their evaluations	63/120 (52.5%)
Diagnostic delay, in years*Median (range)* *n*	From the start of evaluations to age at diagnosis/or/last visit without diagnosis (time of follow-up)	4 (0 to 20) 120
	From the start of evaluations to age at final diagnosis	2 (0 to 15) 45

**Table 2:**
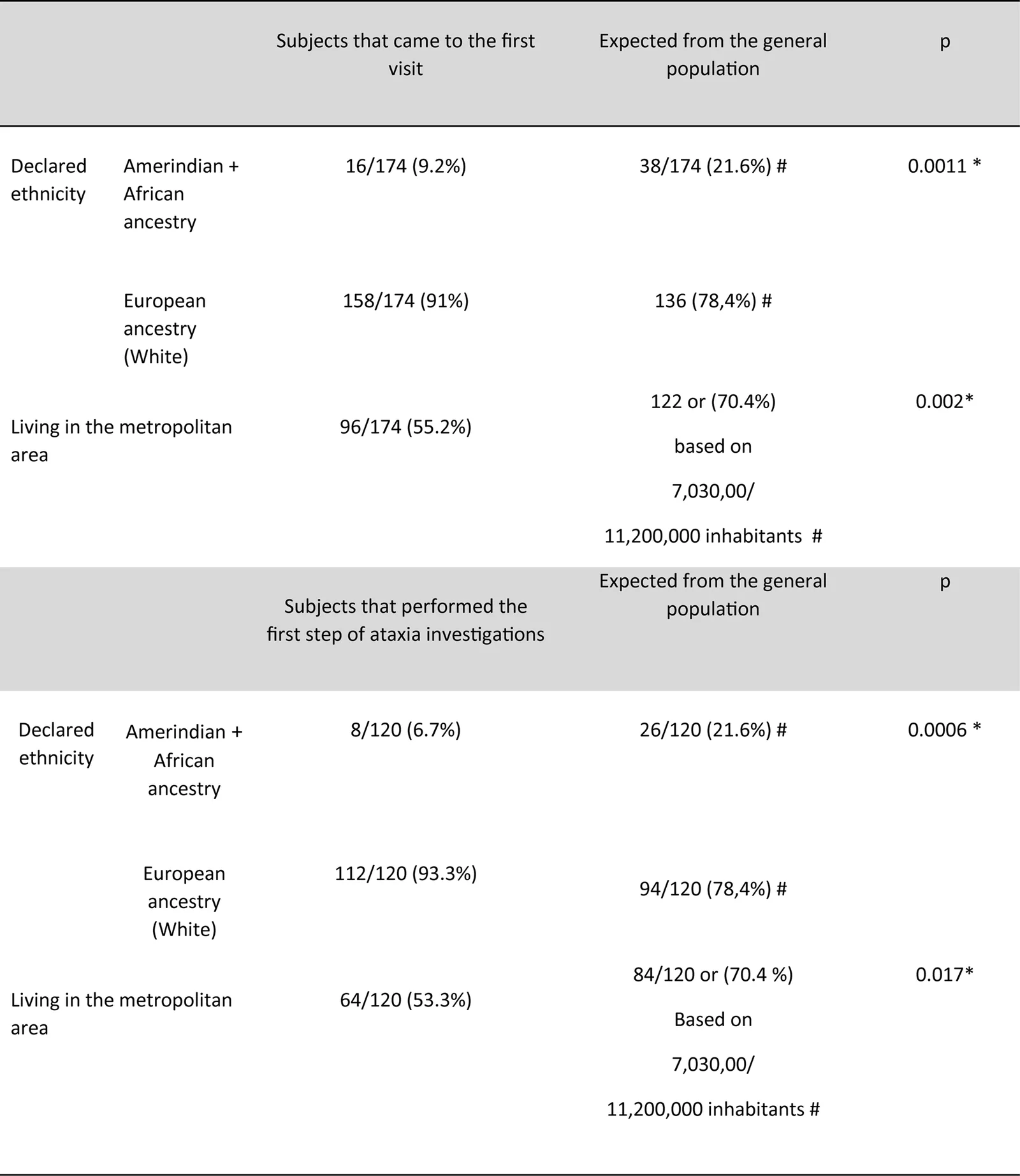
Proportions of the self-declared ethnicity and of the residence origins of the 174 ataxic subjects that attended the first visit and of the 120 that performed the first step of ataxia investigations, compared with the general population of Rio Grande do Sul state, Brazil

* Chi-square

# From: https://www.ibge.gov.br/estatisticas/sociais/saude/22827-censo-demografico-2022.html

### The Main Outcome: to Reach or Not a Final Diagnosis

Among the 120 subjects studied, 63 had an incomplete investigation as their NGS was lacking. From the 57 that had a complete investigation, 45 had a final diagnosis, and 12 did not have it even after an ataxia Sanger panel, exome or genome study.

**Fig. 3:**
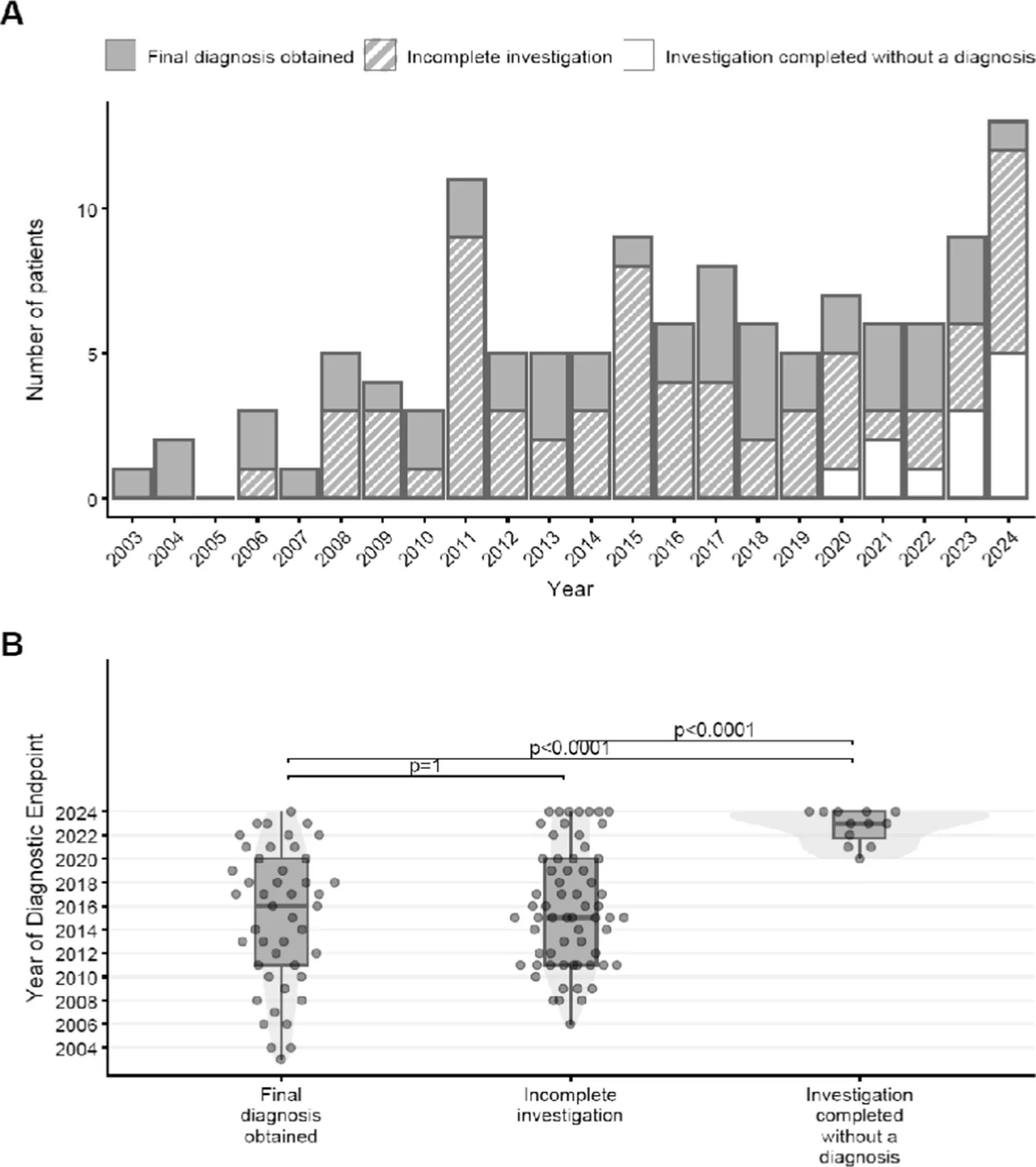
Temporal distribution of ataxic patients according to their outcomes. (**A**) Number of cases with a diagnosis, cases without a diagnosis after a complete investigation, and cases without a diagnosis and without a complete investigation, per year of the end of the diagnostic process or the last follow-up visit. (**B**) The medians (interquartiles) of the chronological years for these same three outcomes

As the year of the first NGS was 2013, this year was used to stratify the cases and reassess the proportions of outcomes before and after that. The results suggested that NGS might have impacted more on the number of subjects that ended their diagnostic investigation without a final diagnosis, than in increasing the relative number of final diagnoses (Table 3). Indeed, the chronological years in which the subjects completed their diagnostic process without a diagnosis were significantly more recent than in the other two outcome groups (Fig. 3). This figure showed that at least half of the subjects characterized as those with pending investigation came in the early years of this cohort, as they stopped coming back to the follow-up visits from 2005 to 2015, many years before the first NGS was actually obtained.

**Table 3:** Proportions of the three main outcomes according to the end of the diagnostic process/last follow-up visit, before or on/after 2013

Year at the end of the diagnostic investigation				
	< 2013	2013 and after	Total	p *
Cases with incomplete investigation	22 (53.7%)	41 (51.9%)	63 (52.5%)	0.119
Cases that finished the evaluation without a diagnosis	1 (2.4%)	11 (13.9%)	12 (10%)	
Cases with a final diagnosis	18 (43.9%)	27 (34.2%)	45 (37.5%)	
Total	41 (100%)	79 (100%)	120 (100%)	

* Chi-square

When patients were stratified by age at onset of the first symptom, a higher proportion of cases with incomplete investigations and a lower proportion of cases with a diagnosis were observed among subjects with an onset age greater than 40 years compared to those with earlier ages at onset (Table 4). Sex, consanguinity between parents, recurrence among siblings and age at the start of investigation were not related to changes in these outcomes (data not shown).

**Table 4:** Proportions of the three main outcomes according to the age at onset of the first symptom

Age at onset of the first symptom						
	< 12 years	Between 12 and 20 years	Between 21 and 40 years	> 40 years	Total	p *
Cases that did not finish their evaluation	18 (42.9%)	8 (34.8%)	12 (52.2%)	25 (83.3%)	63 (51.6%)	
Cases that finished the evaluation without a diagnosis	3 (7.1%)	4 (17.4%)	2 (8.7%)	3 (10%)	12 (9.8%)	0.004 (Linear by linear: 0.0001)
Cases with a diagnosis	21 (50%)	11 (47.8%)	9 (39.1%)	2 (6.7%)	43 (38.5%)	
Total	42 (100%)	23 (100%)	23 (100%)	30 (100%)	118 (100%)	

* Chi-square

Reasons why some ataxic subjects obtained their NGS and finished their diagnostic investigations, and some did not, comprising the “incomplete investigation” group, were explored in the Sect. "Complementary tests" on complementary studies used in this cohort.

### Diagnoses Obtained and the Duration of the Investigation Process

The final diagnoses were FRDA (17 patients, 15 families), A-T (8 patients, 8 families), Coenzyme Q10 deficiency (3 patients, 3 families), NPC (2 patients, 2 families), AOA type 2 (2 patients, 2 families), and SCA2 (2 patients, 2 families); and AOA type 1, Sensory ataxic neuropathy, dysarthria, and ophthalmoparesis due to *POLG* (SANDO-*POLG*), Joubert syndrome, SPG7, SPG 11, SPG76, Short-chain enoyl-CoA hydratase deficiency (ECHS1 deficiency), SCAR 16, Perrault syndrome, Developmental and epileptic encephalopathy (DEE26), and SCA27B, each of them in one individual only. Their clinical characterization was described in the Supplemental material 2, as well as AOfs, ages at start and end of evaluation, delay since the AOfs and since the start until the end of evaluation, year of the last follow-up visit or of the end of the diagnostic evaluation, and method of diagnosis, according to the final status of the 120 subject by 2024.

The median delay between the start of evaluation and the final diagnosis was of 1 (0 to 15) year for the 45 ataxic subjects who obtained a final diagnosis. In-house diagnoses were done in 25 subjects, while the other 20 patients depended on external molecular evaluations (NGS and SCA27B) to have a final diagnosis. The median delay between the start and the end of the diagnostic workout of in-house and external evaluations were of 1 (0 to 9) and 6 (0 to 15) years, respectively (p < 0.0001, Mann–Whitney U test). The external evaluations diagnosed the potentially treatable diseases CoQ10 deficiency, NPC, SANDO-*POLG* and SCA27B many years after onset of symptoms, when instituting a treatment would no longer have the expected effectiveness in most of them. ECHS1 deficiency and Perrault syndrome were another NGS diagnosis whose delays impacted health care, including available screenings and secondary prevention (Supplemental material 2).

### Complementary Tests

#### FXN Analysis

Ninety out of the 120 subjects started their investigation with the *FXN* test as their AOfs was equal or older than 4 years of age. Seventeen of them were molecularly diagnosed as FRDA cases. Taking this as a common denominator, the diagnostic yield of this test would be 18.9%.

To check if the *FXN* test was over-requested by doctors (i.e., in situations where the clinical picture was not compatible), the ninety subjects were stratified into those with and without the three clinical criteria, originally proposed by Anita Harding [21], as a clinical basis for suspecting FRDA: onset of symptoms before the age of 25, progressive unremitting ataxia of the limbs and gait, and absence of the knee and ankle jerks. Forty-one subjects submitted to *FXN* analysis had AO between 4 and 25 years of age: 22/41 and 19/41 had absent and normal reflexes, respectively. Eleven/22 subjects with absent reflexes and 0/19 subjects with normal reflexes carried FRDA. When only the 22 subjects presenting the three clinical criteria were included, the diagnostic yield of *FXN* analysis was 50%.

But FRDA might be presented as a late onset form (LOFA), when the phenotype usually includes pyramidal findings such as brisk reflexes and Babinski sign. Forty-two subjects whose ataxia started after the 25 years of age were tested for *FXN* expansions: 23 with and 19 without brisk reflexes or Babinski sign. Three out of 23 cases with and 1 out of 19 without these pyramidal findings were diagnosed as FRDA.

Mistakes in ordering a *FXN* test included twenty subjects with very early AOfs that performed it, later during their diagnostic process; and three subjects with AOfs older than 48 years, who did not perform it.

#### Alpha-fetoprotein Analysis

Elevated serum concentration of AFP was characterized as the most important blood biomarker in individuals with classic A-T [22, 23]. AFP was performed in 88 out of 120 subjects of the present cohort. All the 27 ataxic subjects with AOfs earlier than 4 years of age started their investigation with this test, while other 61 subjects had it evaluated later in their investigation. AFP was increased in ten (11.3%) subjects: seven were diagnosed with A-T, and two, with AOA2. There was a remaining subject with increased AFP and a clinical picture suggestive of AOA2; his NGS was pending by 2024, and he remained into the incomplete diagnostic group. The negative predictive value of AFP is not 100%, however: the result was normal in one subject (AOfs of 7 years) out of the 8 A-T subjects diagnosed in this cohort.

#### SCAs

Sixty-three subjects performed the main SCA panel, which included *ATXN1, ATXN2, ATXN3, CACNA1A* and *ATXN7* analyses: two of them were identified as having SCA2 (3%). All subjects with AOfs equal or larger than 21 years of age underwent this analysis. The SCA10 assay was incorporated by our institution in 2007: the total number of subjects submitted to the test was smaller—48 cases—and none were carriers.

Finally, DNA samples from 29 subjects with AOfs equal or older than 35 years who remained undiagnosed by 2023 were sent to study GAA repeat expansions in *FGF14*. One subject was found to have SCA27B.

#### Neuroimaging, Metabolic Tests, Neurophysiology

When the initial tests—FXN test, AFP or SCAs panel—showed normal results, the diagnostic process proceeded in a highly individualized manner for each case. In general, however, it included MRI, tests for inborn errors of metabolism, and nerve conduction studies.

Ninety-three ataxic subjects underwent an MRI study: 27/45 with a final diagnosis, 10/12 with a final diagnostic investigation with no diagnosis, and 56/63 cases with an incomplete investigation by 2024.

The MRI was diagnostic for one patient with the malformative Joubert syndrome and detected the ears of the lynx sign in another subject, raising the hypothesis of hereditary spastic paraplegia due to SPG11, later confirmed by a Sanger panel.

Cerebellar atrophy, present in 56 cases, was found in 15 out of 27 subjects with a final diagnosis, 7 out of 10 with a final diagnostic investigation with no diagnosis, and in 34 out 56 cases with an incomplete investigation by 2024.

Linear T2 hypointensities in pons were seen in one A-T carrier and in one subject with an incomplete investigation by 2024. Of note, dentate nucleus hyperintensities were not found in this cohort.

Leukodystrophy was seen in five patients. Only one of them, the SPG11 case, had a final diagnosis. Among the other four, the diagnostic process was completed and negative in one patient, and incomplete in the other three.

Very long chain fatty acids (VLCFA) measured in plasma and the enzymatic activities in peripheral leukocytes of galactocerebrosidase (GALC), arylsulfatase A (ARSA) and hexosaminidase A (HEXA)—biochemical markers of X-linked adrenoleukodystrophy, Krabbe disease, metachromatic leukodystrophy and GM2 gangliosidosis, respectively—were all normal, not only in these cases with leukodystrophy, but also in all the ataxic subjects evaluated with these assays.

Neurophysiological studies of peripheral nerves were obtained in 38 subjects and were found to be abnormal in 15. Mixed axonal-myelinic neuropathies (present in 13 subjects) were the most frequent patterns. Three out of these 15 subjects received a final diagnosis, though not driven by the neurophysiological results: two FRDA and one AOA2.

#### Sanger Panels, Exome and Genome Tests As Final in the Diagnostic Process

Patients in this cohort who remained undiagnosed after the aforementioned tests were referred by their attending physicians for NGS. These studies were considered conclusive by these physicians, establishing that the diagnostic process had been completed.

In the present cohort, the use of tree general types of Sanger Panels was detected: focused on NPC, on hereditary spastic paraplegias (HSPs), and on hereditary ataxias. As cases submitted to panels focused on hereditary ataxias were investigated more comprehensively, their results were of interest in this section.

Targeted Sanger Panel focused on inherited ataxias were done in seven subjects and yielded a molecular diagnosis in five of them. Biallelic pathogenic variants were found in only one allele in the other two subjects, precluding a definitive molecular diagnosis. Exome sequencing was done in seventeen subjects and was diagnostic in nine of them. Genome sequencing was performed in seven individuals—six of whom had previously tested negative on exome or Sanger panels—and only one reached a final diagnosis. Table 3 illustrates how these NGS studies impacted on the main outcome of the present observation, while Table 5 describes the molecular characterization of cases diagnosed via NGS.

**Table 5:** Molecular characterizations of the ataxic subjects diagnosed with Sanger panels—focused on Niemann-Pick type C, on hereditary spastic paraplegias, and on inherited ataxias -, exome and genome sequencings

Diagnosis	ID	Age at onset	Variant	Type	Protein effect	Detected by
Classical ataxia-telangiectasia	TS2928X	1	NM_000051.4(ATM):c.7638_7646del	Deletion In-frame	p.Arg2547_Ser2549del	Exome
			NM_000051.4(ATM):c.3320 T > A	Nonsense	p.Leu1107*	
	JO1404T	1	NM_000051.4(ATM):c.2503del	Nonsense	p.Val835*	Sanger panel for ataxias
			NM_000051.4(ATM):c.5644C > T	Nonsense	p.Arg1882*	
	KJ4136U	2	NM_000051.4(ATM):c.3485 T > G	Nonsense	.Leu1162*	Sanger panel for ataxias
			NM_000051.4(ATM):c.5237G > A	Missense	p.Gly1746Glu	
Coenzyme Q10 deficiency	SQ9019M	3	NM_020247.5(COQ8A):c.1228C > T	Nonsense	p.Arg410*	Sanger panel for ataxias
			NM_020247.5(COQ8A):c.1228C > T	Nonsense	p.Arg410*	
	QS9027L	1	NM_020247.5(COQ8A):c.895C > T	Missense	p.Arg299Trp	Exome
			NM_020247.5(COQ8A):c.1706C > A	Missense	p.Ala569Asp	
	MC7673O	11	NM_020247.5(COQ8A):c.1042C > T	Nonsense	p.Arg348*	Exome
			NM_020247.5(COQ8A):c.1013C > T	Missense	p.Ala338Val	
Niemann-Pick type C	GF6972R	31	NM_000271.5(NPC1):c.3019C > G	Missense	p.Pro1007Ala	Sanger panel for NPC
			NM_000271.5(NPC1):c.3019C > G	Missense	p.Pro1007Ala	
	MY3080G	12	NM_000271.5(NPC1):c.3019C > G	Missense	p.Pro1007Ala	Sanger panel for NPC
			NM_000271.5(NPC1):c.3246-10_3246-8del	Acceptor splice site		
AOA2*	GI7916P	14	NM_015046.7(SETX):c.5308_5311del	frameshift	p.Glu1770Ilefs*15	Sanger panel for ataxias
			NM_015046.7(SETX):c.5308_5311del	frameshift	p.Glu1770Ilefs*15	
	AL7063X	14	NM_015046.7(SETX):c.994_1994del	Deletion In-frame	Protein length changes	Sanger panel for ataxias
			NM_015046.7(SETX):c.470A > G	Missense	p.Tyr157Cys	
AOA1*	VJ4760T	3	NM_001195248.2(APTX):c.837G > A	Nonsense	p.Trp279*	Exome
			NM_001195248.2(APTX):c.837G > A	Nonsense	p.Trp279*	
SANDO-*POLG* **	IT5420Y	19	NM_002693.3(POLG):c.2243G > C	Missense	p.Trp748Ser	Exome
			NM_002693.3(POLG):c.2243G > C	Missense	p.Trp748Ser	
Hereditary Spastic Paraplegia 7	DH2546Q	36	NM_003119.4(SPG7):c.1529C > T	Missense	p.Ala510Val	Sanger panel for HSPs
			NM_003119.4(SPG7):c.1460A > G	Missense	p.Glu487Gly	
Hereditary Spastic Paraplegia 11	JE5045S	12	NM_025137.4(SPG11):c.4790G > A	Nonsense	p.Trp1597*	Sanger panel for HSPs
			NM_025137.4(SPG11):c.1825C > T	Nonsense	p.Gln609*	
Hereditary Spastic Paraplegia 76	XX7593F	30	NM_005186.4(CAPN1):c.618_619del	Frameshift	p.Gly208Glnfs*7	Genome
			NM_005186.4(CAPN1):c.618_619del	Frameshift	p.Gly208Glnfs*7	
ECHS1 *** deficiency	SP9803S	2	NM_004092.4(ECHS1):c.713C > T	Missense	p.Ala238Val	Exome
			NM_004092.4(ECHS1):c.3G > C	Initiation codon	p.Met1?	
SCAR16 ^#^	VR5358B	27	NM_005861.4(STUB1):c.786 + 1G > A	Canonical donor splice site		Exome
			NM_005861.4(STUB1):c.433A > C	Missense	p.Lys145Gln	
Perrault Syndrome Type 1	QX1579B	10	NM_000414.4(HSD17B4):c.1369A > T	Missense	p.Asn457Tyr	Exome
			NM_000414.4(HSD17B4):c.439A > T	Missense	p.Ile147Phe	
DEE26 ^##^	SB8148Z	1	NM_004975.4(KCNB1):c.1184G > A	Missense	p.Gly395Glu	Exome

*AOA: ataxia with oculomotor apraxia; ** SANDO-*POLG*: Sensory ataxic neuropathy, dysarthria, and ophthalmoparesis due to POLG; ***ECHS1: Short-chain enoyl-CoA hydratase; ^#^ SCAR16: Autosomal recessive spinocerebellar ataxia type 16; ^##^ DEE26: Developmental and epileptic encephalopathy-26

As previously described, 63 cases had incomplete investigations at the end of the observation period due to not obtaining an NGS. These patients were compared to the 29 subjects who underwent NGS testing—regardless of whether these 29 subjects have had a diagnosis. Patients with and without access to NGS had their last follow-ups in 2022 (2013 to 2024) and 2015 (2006 to 2024) (p = 0.0001, MW U test). The proportions of sex, schooling (for those with 21 years or more), and living or not in the metropolitan area were similar between groups (data not shown). Patients with and without access to NGS had median (IQR) AOfs of 12 (25) and 27 (39) years (p = 0.003, MW U test), and ages at first evaluation of 17 (25) and 36 (37) years (p = 0.010, MW U test).

## Discussion

This study revealed that 174 patients with ataxia—with an onset of up to one year prior and no vertical family history—were referred to our service for genetic evaluation between 2003 and 2021. These figures correspond to 9.4 cases per year per 11 million inhabitants in the Brazilian state of Rio Grande do Sul. A body of evidence pointed to obstacles in their access to health services. First, many ataxic subjects (54 or 30%) were lost immediately after the first consultation. Secondly, there were smaller-than-expected proportions of Black and Amerindian participants, ethnic groups associated with social minorities in our country. Thirdly, sixty-three ataxic subjects did not undergo an NGS by the end of the study, in 2024. These figures reflect the difficulties patients and healthcare teams faced over the past 20 years in reaching a diagnosis. Social disparities — loss to follow-up visits and racial discrimination —and the unavailability of NGS within the SUS appear to be among the main obstacles to the diagnostic process revealed by this cohort.

In contrast, fifty-seven subjects finished their investigation either because a diagnosis was achieved—in 45 of them—or because their NGS did not find any causal mutation. Even for those individuals with access to NGS, its absence within the public health system resulted in a consequent delay in obtaining the test, diagnosis, and management, where available. This was the case of the potentially treatable diseases CoQ10 deficiency, NPC, SANDO-*POLG* and SCA27B, and the potentially manageable diseases ECHS1 deficiency and Perrault syndrome.

We did not feel encouraged to estimate the diagnostic frequencies through this retrospective review. The subjects included were investigated by different clinical teams across a large period of time. Their clinicians did not necessarily follow standardized protocols; however, the number of complementary tests ordered and the long follow-up—with a median of four years (Table 1)—reflected the effort invested in seeking the diagnosis of these patients. A few clinical mistakes were identified, such as the inappropriate use of diagnostic tests (*FXN* analyses in patients with AOfs between zero and three years), or their lack (the same analysis in few people with AOfs over 40 years). As in any long-term retrospective cohort, several methodological limitations prevented us from inferring relative frequencies of diagnoses for the background population. HA are rare diseases and any additional or absent case might distort frequencies, minimal incidence or prevalent rates. Despite this, the most common forms found were very consistent with what has been described in other studies from Latin America and some other regions of the world [15, 24–27]. If the 174 subjects who came to a first visit were indeed hereditary ataxia cases, this figure would represent 1.6 cases of non-vertical ataxias per 100,000 local inhabitants. If we add the local prevalence of the most common SCA in Rio Grande do Sul, SCA3/MJD, estimated to be 7 per 100,000 [28], the overall prevalence of HA in this Brazilian state would be similar to numbers obtained elsewhere [29]. Despite this, we still think that the non-vertical ataxias were underestimated in the present report.

We found higher-than-expected proportions of white subjects and subjects living in the countryside of our state. Although Black and mixed-race persons are less frequent in these regions [30], this was not enough to explain their reduced number in our cohort. A possible explanation for the low number of Black or Amerindian individuals would be a selection bias in healthcare referrals related to race [31] and low socioeconomic status [32].

In contrast, we did not detect any selection bias favouring the reference of ataxic patients living in small communities, outside the metropolitan area. This finding may reflect a truly biological event, such as higher inbreeding rates in small cities and rural populations. Our finding suggests that inbreeding in different populations of our region deserves further investigation.

Most diagnoses were obtained by molecular tests, except for one MRI diagnosis of Joubert syndrome and for five cases whose clinical picture plus complimentary studies were considered diagnostic of classical A-T. The yields for the first step diagnostic tools were 50% for GAA analysis of *FXN* (11/22 cases presenting the three classical criteria), 11.3% for AFP (10/88), 4.8% for the SCA investigation—the panel for SCA1, SCA2, SCA3/MJD, SCA6, SCA7, and SCA10, followed by the direct test for SCA27B (3/63) -, and 1% for MRI (1/93). The yields for the final steps studies were 71.4% for Sanger panels focused on ataxias (5/7), 52.9% for exomes (9/17), and 14% for genomes (1/7). Although listed here, these yields should not be directly compared, as each test was indicated for different patient profiles and groups. One good example was the number of MRI studies per final outcome: the number was proportionally lower among cases with a final diagnosis because positive molecular analyses for FRDA and SCAs were obtained before doctors deemed it necessary to order neuroimaging. The importance of the MRI example continues: it detected three cases of leukodystrophy that remained unsolved in 2024 due to the lack of NGS. In contrast, neurophysiological studies did not appear to be of any help in investigating the present ataxia cases.

Four of the 45 diagnosed cases carried autosomal dominant mutations: the two SCA2 cases, the SCA27B patient, and the case with DEE26. With the exception of the last one—who carried a *de novo* mutation and was severely affected by the disease —the other subjects´ relatives were able to benefit from genetic counseling and the option of predictive testing. The largest group comprised individuals with autosomal recessive conditions—or 41 participants. Thirteen had recurrence among sibs (nine cases of FRDA, the two NPC subjects, one case of CoQ10 deficiency, and the SPG7 patient), while the other 28 patients —the majority of those with recessive forms—were the only affected individuals in their sibships (Supplemental materials 2 and 3). This finding underscores the importance of maintaining a clinical suspicion of HA and of proceeding with molecular investigation even in the absence of a family history.

An important weakness of this cohort was the non-availability of analyses of repeat expansions in the second intron of the replication factor complex subunit 1 (*RFC1*) gene, for the diagnosis of cerebellar ataxia, neuropathy, and vestibular areflexia syndrome (CANVAS). CANVAS is characterized by a late, slowly progressive sensory neuropathy, cerebellar dysfunction, and vestibular impairment [33], and is one of the most common adult-onset HA in many populations [34–36]. We can anticipate the undiscovered existence of CANVAS cases in our cohort, and we hope to address this gap soon.

Despite the cautious comments above, the diagnostic yields presented here might help to think about the most effective diagnostic protocol for the ataxia disorders found in our population. GAA analysis of *FXN* in general, in this cohort, had a relatively low diagnostic yield (18.9%) because they were requested too widely, to patients without a FRDA clinical picture. We interpreted this fact as resulting from the availability of the test at our hospital while other more suitable tests were unavailable. In contrast, Sanger, exome and genome panels had better diagnostic yields even than SCA panels, among individuals with onset after 20 years of age. Of course, the analysis of the adult-onset cases was hampered by the unavailability of *RFC1* analyses. However, the number and proportion of diagnoses among adult-onset ataxias obtained by NGS plus other Sanger panels (NPC, SANDO-*POLG*, SPG7, SCAR16, and SPG76, one case each, or 5/8 individuals tested) were larger than those detected by SCA panels (two SCA2 and one SCA27B, or 3/53 individuals tested). Because of that, we propose that the molecular studies of a diagnostic protocol for non-vertical ataxias in our population should follow the order: *FXN*, then NGS, then the analyses of other diseases caused by expanded repeats.

In contrast to the lack of access to NGS and *RFC1* studies, the present cohort had access to other extensive investigations such as neuroimaging, neurophysiology, and IEM studies. As previously described, only one out of 96 (1%) MRIs was diagnostic (the Joubert syndrome case). MRI was lacking in two out of 63 cases with incomplete investigations, so we are not sure if it could have helped their diagnoses. In contrast, hundreds of studies for IEM—organic acids, amino acids, VLCFA, ARSA, HEXA, GALC, ceruloplasmin, etc.—gave normal results, and the 38 neurophysiologic studies did not help much in focusing the diagnosis. These results deserve serious reflection, from the point of view of health policies. We are not saying that the IEM should not be in the main hypotheses for the diagnostic investigation of ataxic patients, but that the diagnostic yield of the biochemical tests for the ataxia setting is low. IEM, if present, could be detected by NGS.

The lack of NGS in the SUS explains not only the large number of unsolved Brazilian cases to date, but also the unnecessary diagnostic costs related to the care of patients with ataxia, as physicians may be compelled to continue carrying out a least promising investigation line in the absence of more effective ones. A third and alarming effect of the difficulty in obtaining NGS is the extremely long diagnostic delays for those that wait for these tests (Supplemental material 2). A delayed diagnosis means delayed genetic counselling and delayed treatment of carriers. The healthcare delay of carriers of potentially treatable diseases CoQ10 deficiency, NPC, SANDO-POLG and SCA27B of the present cohort was longer than those measured in SCAs and FRDA of our region [37, 38]. SANDO-*POLG* and SCA27B of the present cohort was longer than those measured in SCAs and FRDA of our region.

While NGS and specific studies for CANVAS and SCA27B are lacking at our institution, the situation is even worse in most public ataxia clinics in our country, where investigations such as the GAA test for FRDA and the SCA panel are unavailable. Patients treated at these clinics must seek these diagnoses through private examinations, which likely results in access difficulties and social disparities far greater than those observed in Rio Grande do Sul state.

## Conclusions

If recommendations regarding diagnostic protocols can be drawn from our 20 years of experience with ataxias without a vertical history, one of them is that each institution should develop its own, just as we have now done for ourselves. The availability of in-house diagnostic studies must be among the primary determinants of a protocol to be used in public health systems. But this is not enough: a better diagnostic protocol for these non-vertical ataxias will be established when GAA study in *FXN*, gene panel/exome/genome sequencing and SCA panels are truly available to everyone. Based on the diagnostic yields identified here, we suggest to establish, as soon as possible, a diagnostic protocol for non-vertical ataxias in the following order: *FXN*, then NGS, then the analyses of other diseases caused by expanded repeats.

In summary, we found that FRDA, classical A-T, Coenzyme Q10 deficiency, NPC, AOA2 and SCA2 were the most common HA manifested without a vertical family history in our population; other even rarer conditions were also present. Reduced access to HA investigation occurred, causing disparities especially affecting Black and Amerindian individuals, and delays in the start of specific treatments. A diagnostic protocol is needed that retains currently available tests—AFP, MRI, GAA study in *FXN*, and SCA panel -, and includes the investigation of novel repeat expansions associated with ataxias and NGS-based testing—exome sequencing being the first choice—in order to expand access to diagnosis and genetic counselling, as well as to improve the management of these patients.

## Supplementary Information

Below is the link to the electronic supplementary material.

Supplementary file1 (DOCX 12 KB)

Supplementary file2 (DOCX 454 KB)

Supplementary file3 (XLSX 92 KB)

## Data Availability

The research data is available as the Supplemental Material 3, submitted together with the main manuscript.
