# Supplementary material for: The Real Life of Ataxia Patients Without a Vertical Family History: a Twenty-year Experience in South Brazil
Source: Cerebellum. 2026 Aug 19;25(5):128. doi: 10.1007/s12311-026-02067-2 (PMC13490217; doi:10.1007/s12311-026-02067-2)
Supplement: Supplementary file 1 [file 12311_2026_2067_MOESM1_ESM.docx]

**Supplemental material 1**

**Variables collected for all patients included in the study, encompassing demographic, clinical, neuroimaging, biochemical, molecular data, and outcomes**

**Demographic data:**

| Gender | Male / Female |
| --- | --- |
| Date of Birth | Day / Month / Year |
| Year of Birth | Four-digit year |
| City of Birth |  |
| City of Residence |  |
| Year of onset of investigation | Four-digit year |

**Clinical data:**

| Index Case | Yes / No/not registered |
| --- | --- |
| Year of First Symptom | Year when the first symptom appeared |
| Age of Onset (first symptom) | Age in years at symptom onset |
| AO Gait Ataxia | Age of onset of gait ataxia/not registered |
| First Symptom | Description of initial symptom |
| Parental Consanguinity | Yes / No/not registered |
| Recurrence Among Siblings | Yes / No/not registered |
| Year of Physical Exam | Year of clinical evaluation |
| Cognitive Loss | Yes / No/not registered |
| Recent Memory Deficit | Yes / No/not registered |
| Gait Ataxia | Yes / No/not registered |
| Dysmetria | Yes / No/not registered |
| Dysdiadochokinesia | Yes / No/not registered |
| Dysarthria | Yes / No/not registered |
| Oculomotor Apraxia | Yes / No/not registered |
| Nystagmus | Yes / No/not registered |
| Nuclear Ophthalmoparesis | Yes / No/not registered |
| Supranuclear Ophthalmoparesis | Yes / No/not registered |
| Slow Saccadic Eye Movements | Yes / No/not registered |
| Broken-Up Pursuit | Yes / No/not registered |
| Square Wave Jerks | Yes / No/not registered |
| Paresis of the Lower Limbs | Yes / No/not registered |
| Paresis of the Upper Limbs | Yes / No/not registered |
| Spasticity | Yes / No/not registered |
| Hyperreflexia | Yes / No/not registered |
| Babinski Sign | Yes / No/not registered |
| Ankle Clonus | Yes / No/not registered |
| Extrapyramidal Symptoms | Yes / No/not registered |
| Amyotrophy | Yes / No/not registered |
| Areflexia of the Lower Limbs | Yes / No/not registered |
| Vibration Hypoesthesia | Yes / No/not registered |
| Hypoalgesia | Yes / No/not registered |
| Thermal Hypoesthesia | Yes / No/not registered |
| Dysphagia | Yes / No/not registered |
| Scoliosis | Yes / No/not registered |
| High-Arched Foot | Yes / No/not registered |
| Optic Atrophy | Yes / No/not registered |
| Hearing Loss | Yes / No/not registered |
| Seizures | Yes / No/not registered |
| Urinary Incontinence | Yes / No/not registered |
| Erectile Dysfunction | Yes / No/not registered |

**Electroneurophysiological data:**

| Electromyoneurography | Performed (Yes/No) |
| --- | --- |
| Year of Electromyoneurography | Four-digit year |
| Neuropathy Type (motor/sensory/sensorimotor) | 0 = normal; 1 = motor; 2 = sensory; 3 = sensorimotor |
| Neuropathy Pathophysiology | 0 = normal; 1 = demyelinating; 2 = axonal; 3 = mixed |

**Neuroimaging data:**

| Brain MRI | Performed (Yes/No) |
| --- | --- |
| Year of Brain MRI | Four-digit year |
| Cerebellar Atrophy on MRI | Yes / No |
| Leukodystrophy on MRI | Yes / No |
| Pontine/Cerebellar Peduncle T2 Hypointensities | Yes / No |
| Dentate Nucleus Hyperintensity | Yes / No |
| Other | Yes / No |

**Biochemical data:**

|  |  | Result |
| --- | --- | --- |
| Serum α-Fetoprotein > 7 ng/mL | Yes / No |  |
| Hexosaminidase A Deficiency | Yes / No |  |
| Hexosaminidase B Deficiency | Yes / No |  |
| Arylsulfatase A Deficiency | Yes / No |  |
| Presence of Sulfatides in Urine | Yes / No |  |
| Galactocerebrosidase Deficiency | Yes / No |  |
| Elevated Very Long Chain Fatty Acids | Yes / No |  |
| Total Cholesterol < 40 mg/dL | Yes / No |  |
| Triglycerides < 0.20 g/L | Yes / No |  |
| Phytanic Acid | Yes / No |  |
| Plasma α-Tocopherol < 4 µmol/L | Yes / No |  |
| Serum ceruloplasmin | Yes / No |  |

**Molecular data:**

|  |  | Result |
| --- | --- | --- |
| *FXN* GAA Expansion | Performed (Yes/No) | 1 = monoallelic; 2 = biallelic |
| *FXN* SNV | Performed (Yes/No) |  |
| SCA1 Molecular Testing | Performed (Yes/No) |  |
| SCA2 Molecular Testing | Performed (Yes/No) |  |
| SCA3 Molecular Testing | Performed (Yes/No) |  |
| SCA6 Molecular Testing | Performed (Yes/No) |  |
| SCA7 Molecular Testing | Performed (Yes/No) |  |
| SCA10 Molecular Testing | Performed (Yes/No) |  |
| SCA12 Molecular Testing | Performed (Yes/No) |  |
| SCA17 Molecular Testing | Performed (Yes/No) |  |
| DRPLA Molecular Testing | Performed (Yes/No) |  |
| FXTAS Molecular Testing | Performed (Yes/No) |  |
| Sanger Panel targeted on SPG | Performed (Yes/No) |  |
| Sanger panel targeted on NPC |  |  |
| *RFC1* AAGGG Expansion | Performed (Yes/No) |  |
| *FGF14* GAA Expansion | Performed (Yes/No) |  |
| Sanger panel targeted on ataxias (NGS) | Performed (Yes/No) |  |
| Exome Sequencing (NGS) | Performed (Yes/No) |  |
| Genome Sequencing (NGS) | Performed (Yes/No) |  |

**Outcomes**

| Final status in 2024 | 1=Incomplete investigation; 2=investigation completed (NGS was done) without a diagnosis; 3=Diagnosis was determined |
| --- | --- |
| Diagnosis |  |
| Diagnostic delay since AOfs |  |
| Diagnostic delay since the start of investigation |  |
