# Supplementary material for: The Real Life of Ataxia Patients Without a Vertical Family History: a Twenty-year Experience in South Brazil
Source: Cerebellum. 2026 Aug 19;25(5):128. doi: 10.1007/s12311-026-02067-2 (PMC13490217; doi:10.1007/s12311-026-02067-2)
Supplement: Supplementary file 2 [file 12311_2026_2067_MOESM2_ESM.docx]

**Supplemental material 2**

**from Aschoff et al, “The real life of ataxia patients without a vertical family history: a twenty-year experience in South Brazil”**

**Summary**

|  | Page |
| --- | --- |
| 1. Figure - General results from the algorithm used to define the main outcomes | 3 |
| 2. Table - The final status of the diagnostic workout of the present 120 ataxic patients under study | 4 |
| 3. Clinical characterization of the positive diagnoses | 5 |
| 3.1 Friedreich ataxia (FRDA) | 5 |
| 3.2 Ataxia-telangiectasia | 6 |
| 3.3 Coenzyme Q10 deficiency (CoQ10 deficiency) | 6 |
| 3.4 Niemann Pick type C (NPC) | 7 |
| 3.5 Spinocerebellar Ataxia type 2 (SCA2) | 7 |
| 3.6 Ataxia-Oculomotor Apraxia 2 (AOA2) | 8 |
| 3.7 Ataxia-Oculomotor Apraxia 1 (AOA1) | 8 |
| 3.8 Sensory ataxic neuropathy, dysarthria, and ophthalmoparesis due to *POLG* (SANDO-*POLG*) | 9 |
| 3.9 Joubert syndrome | 9 |
| 3.10 Spastic paraplegia 7 (SPG7) | 9 |
| 3.11 Spastic paraplegia 11 (SPG11) | 9 |
| 3.12 Spastic paraplegia 76 (SPG76) | 9 |
| 3.13 Short-chain enoyl-CoA hydratase (ECHS1) deficiency | 10 |
| 3.14 Autosomal recessive spinocerebellar ataxia type 16 (SCAR 16) | 10 |
| 3.15 Perrault syndrome | 10 |
| 3.16 Developmental and epileptic encephalopathy-26 (DEE26) | 11 |
| 3.17 Spinocerebellar Ataxia 27B (SCA 27B) | 11 |

1. **Figure - General results from the algorithm used to define the main outcomes of the study**

2. **Table - The final status of the diagnostic workout of the present 120 ataxic patients under study.**

Aggregate data about years were presented in median (range)

| Final status | Number of cases | Number of families | Age at onset of the first symptom | Age at the start of evaluation | Age at the end of evaluation | Delay since start of evaluation | Year of the last follow-up visit or of the end of the diagnostic evaluation | Delay between the end of evaluation and AOfs | Diagnosis made through |
| --- | --- | --- | --- | --- | --- | --- | --- | --- | --- |
| Incomplete diagnostic evaluation | 63  (52.5%) | 62 | 27 (0 to 67) | 36 (4 to 68) | 42 (6 to 75) | 4 (0 to 20) | 2015 (2006 to 2024) | 8 (1 to 21) | - |
| No diagnosis at the end of diagnostic evaluation | 12 (10%) | 12 | 15 (0 to 60) | 21.5 (6 to 61) | 27.5 (15 to 75) | 10 (4 to 15) | 2023 (2020 to 2024) | 15.5 (7 to 19) | - |
| With a final diagnosis | 45  (37.5%) | 43 | 12 (0 to 65) | 17 (2 to 76) | 21 (2 to 83) | 1 (0 to 15) | 2016 (2003 to 2024) | 9 (0 to 19) | - |
| Friedreich ataxia | 17 (14.17%) | 15 | 18 (5 to 40) | 23 (9 to 50) | 23 (9 to 50) | 0 (0 to 7) | 2012 (2003 to 2021) | 7 (0 to 16) | Direct analysis of GAA repeats |
| SCA 2 | 2 (1.7%) | 2 | 40 (30 and 50) | 51 (43 and 59) | 52 (44 and 60) | 1 (0 and 2) | 2015 (2012 and 2017) | 12 (9 and 15) | Direct analysis of CAG repeats |
| Ataxia telangiectasia | 8 (6.7%) | 8 | 2.5 (1 to 7) | 7 (2 and 9) | 7.5 (2 to 11) | 1 (0 to 5) | 2010 (2006 to 2021) | 5 (1 to 8) | Clinical and Biochemical |
| NPC | 2 (1.7%) | 2 | 21.5 (12 and 31) | 31 (26 na 37) | 34 (27 and 41) | 2.5 (1 and 4) | 2015 (2013 and 2017) | 12.5 (10 and 15) | Target gene sequencing and biochemical |
| AOA 2 | 2 (1.7%) | 2 | 14 (14 and 14) | 20 (18 and 22) | 25 (21 and 29) | **4.5 (3 and 6)** | 2017 (2016 and 2018) | 10.5 (7 and 14) | Target gene Sequencing |
| POLG | 1 (0.84%) | 1 | 19 | 22 | 28 | 6 | 2015 | 9 | Exome Sequencing |
| Joubert syndrome | 1 (0.84%) | 1 | 0 | 10 | 12 | 2 | 2013 | 11 | Clinical and Radiologic |
| Coenzyme 10 deficiency | 3 (2.5%) | 3 | 3 (1 to 11) | 5 (5 to 17) | 20 (19 to 28) | **14 (11 to 15)** | 2022 (2020 and 2023) | 18 (17 to 18) | Exome Sequencing |
| SPG 11 | 1 (0.84%) | 1 | 12 | 13 | 23 | 9 | 2018 | 12 | Panel Sequencing |
| ECHS1 deficiency | 1 (0.84%) | 1 | 2 | 8 | 11 | 3 | 2022 | 9 | Exome Sequencing |
| SPG 7 | 1 (0.84%) | 1 | 36 | 42 | 46 | 4 | 2020 | 10 | Panel Sequencing |
| SCAR 16 | 1 (0.84%) | 1 | 27 | 28 | 39 | 11 | 2022 | 12 | Exome Sequencing |
| Perrault syndrome | 1 (0.84%) | 1 | 10 | 12 | 25 | 13 | 2023 | 15 | Exome Sequencing |
| AOA 1 | 1 (0.84%) | 1 | 3 | 11 | 19 | 9 | 2018 | 16 | Exome Sequencing |
| Developmental and epileptic encephalopathy *KCNB1* | 1 (0.84%) | 1 | 1 | 6 | 13 | 7 | 2023 | 13 | Exome Sequencing |
| SCA 27B | 1 (0.84%) | 1 | 65 | 76 | 83 | 8 | 2024 | 19 | Direct analysis of GAA repeats |
| SPG 76 | 1 (0.84%) | 1 | 30 | 38 | 48 | 10 | 2021 | 17 | Genome Sequencing |
| All | 120  (100%) | 117 | 18.5 (0 to 67) | 23 (2 to 76) | 27 (2 to 83) | 4 (0 to 20) | 2016 (2003 to 2024) | 9 (0 to 21) |  |

Of note, in-house diagnoses included 17 FRDA, 2 SCA2, 2 classical A-T whose diagnoses were clinical, one Niemann-Pick type C (NPC), one Joubert syndrome, one hereditary spastic paraplegia (from now, specific hereditary spastic paraplegias were identified by the name of their genes) due to *SPG7* mutation (SPG7), and one SPG11.

3. **Clinical characterization of the positive diagnoses**

**3.1 Friedreich ataxia (FRDA)**

The 17 FRDA cases belonged to 15 independent families. Fourteen patients have been already reported [39] (Fussiger et al 2019); the other three were diagnosed between 2018 and 2021. Ataxia, absent reflexes and first symptoms starting before 25 years of age were present in 13/17 (76.4%); although Babinski sign was not registered in two, we assumed that these patients had the classical phenotype. The other four patients (23.6%) had late onset FRDA (LOFA): two were siblings and presented FRDA with retained reflexes (FARR).

All patients carried GAA expansions in their two alleles. The median (range) diagnostic delay between the start and end of evaluation was one (zero to one) year in 16 cases: the remaining patient was identified only five years after starting his evaluation. He was the index case of the FARR sibship mentioned above. Ataxic since his 40 years of age, he presented with gait and limb ataxia, dysarthria, brisk reflexes with ankle clonus, Babinski sign, reduced vibratory sensibility, dysphagia and reduced vision. An initial hypothesis of SCA7 directed the investigation to the SCA panel. Technical problems with his DNA extraction and several missing of follow-up visits appeared to have been the main causes of the delayed diagnosis in this case. Her affected sister came after him and experienced a more typical diagnostic delay.

**3.2 Ataxia-telangiectasia**

The eight classical A-T of the present cohort started their ataxic manifestations between 1 and 7 years of age. Oculomotor apraxia/nystagmus/slow saccades, extrapyramidal findings and telangiectasias were registered in 6, 4, and 7 cases. Classical A-T was diagnosed based on the clinical presentation plus the presence of at least two of the following findings: high levels of AFP, increased chromosomal breaks, and humoral immunodeficiency. High levels of AFP were detected in seven cases; the subject without high AFP started his manifestations at 7 years of age and presented increased chromosomal breaks and decreased serum immunoglobulin A levels.

The diagnostic delay between the start of evaluation and clinical diagnosis varied from zero to two years in seven cases. Diagnosis was delayed five years in the eighth case due to a previous long investigation centered in the suspicion of a fatty acid beta-oxidation defect.

In 2021 and 2022, exome sequencings were obtained for A-T subjects MY3080G, BR3245G and BW8311B who started their evaluations in 2009, 2019 and 2021, respectively. All were compound heterozygotes and their molecular information were presented in **Table 5** of the main manuscript.

**3.3 Coenzyme Q10 deficiency (CoQ10 deficiency)**

Three index cases with onset of symptoms between 1 and 11 years of age were diagnosed with CoQ10 deficiency. Their molecular information was presented in **Table 5** of the main manuscript.

Case GA8509Z was a boy from consanguineous parents who started at 3 years of age with a very slow progressive gait ataxia; two sibs had similar symptoms. Followed by annual visits since 2007, his neurologic examination by the time of diagnosis, at 20 years of age in 2022, disclosed normal cognition, limb and axial ataxia with slight difficulties in walking, bradykinesia, ankle clonus, and gaze-evoked nystagmus. He obtained an order from the Judiciary for the State to provide a Sanger panel that established his diagnosis. He started receiving oral coenzyme Q10 (CoQ10) 750mg/day in 2022, when his Scale of Assessment and Rating of Ataxia (SARA) [40] (Schmitz-Hübsch et al 2006) scored 4.5. In the last follow-up visit, in 2024, at 22 years of age, his SARA was 11.

Case AO1111A was a boy who lost walking abilities at 2 and started investigating with us at 6 years of age. Gait and limb ataxia, dysarthria, slow saccades and restriction of vertical eye movements were the present at 6, plus generalized seizures and a mild cognitive decline since 12 years of age. A private exome was arranged by the family at 19 years of age, in 2021, when he started replacement therapy with oral CoQ10 400 mg 2x/day (10 mg/Kg/day). He lost follow-up visits in 2022, at 20 years of age.

Case BZ6781A was also a boy who started symptoms and investigation at 11 and 17 years of age, respectively. At first visits, he presented with gait and limb ataxia, dysarthria, and keratoconus. SARA scored 8, 8, 10, 9,10, 12.5, 7.5 and 8 points when he was 21, 22, 23, 24, 25, 27, 28 and 30 years-old. Seizures started by age 22, in 2017, and were easily controlled with valproate. A Sanger panel for ataxia genes was negative in 2022. Exome sequencing detected mutations at *COQ8A* in 2023; Coq10 replacement therapy was then started with 500 mg/day (17 mg/kg/day).

**3.4 Niemann Pick type C (NPC)**

Two index cases were suspected and later diagnosed as having NPC. Their molecular information was presented in **Table 5** of the main manuscript.

Case WN4711A was a woman who started symptoms and investigation at 31 and 39 years of age, respectively. With consanguineous parents and one sister with similar findings, she complained of auditory hallucinations and presented with gait and limb ataxia, and restriction of vertical eye movements. Two years later, she developed hypophonia, dysphagia, dystonic posture of the trunk, bradykinesia, ankle clonus and absent vertical saccades. Her Filipin test was negative, but a Sanger panel sequencing for lysosomal diseases found a homozygous mutation at *NPC1*. She began receiving Miglustat at the age of 42. At 45, in 2017, she continued deteriorating. She was wheelchair bound, developed spasticity, a complete ophthalmoplegia and dull mood, and was lost from follow-up visits.

Case DI8215S was a man whose symptoms and investigation started when he was 12 and 26 years of age, respectively. Relatives reported a progressive loss of cognitive abilities and of coordination that prevented running. At neurologic examination, there was gait and limb ataxia, action tremor, dysarthria, bradykinesia, loss of down vertical saccades, cervical dystonia and spasticity in lower limbs. The Filipin test was inconclusive and a Sanger panel sequencing for lysosomal diseases found mutations at *NPC1* at age 27. He started Miglustat at 28 years of age, in 2018, and continued using it by 2024, with an apparently stable clinical picture.

**3.5 Spinocerebellar Ataxia type 2 (SCA2)**

Two subjects were diagnosed with SCA2.

The first was a man that started symptoms and investigation when he was 40 and 43 years of age. Without a positive family history, he presented with gait and limb ataxia, slow saccades and brisk reflexes. The SCA panel performed when he was 44 years of age detected 22/42 CAG repeat alleles at *ATXN2*. After receiving his diagnosis, he reported that his father had parkinsonian manifestations by the age of 73 and soon died after this, many years ago. One of his two children participated in the Predictive Genetic Testing Program of our institution. He lost follow-up visits in 2021, at 47 years of age.

The second case was a woman without children that started with gait ataxia at 50 years of age. She started her evaluation by age 59, reporting that two sisters but not her ancestors presented similar symptoms. At examination, she presented a depressive mood, gait and limb ataxia, nystagmus, slow saccades, blepharospasm, orthostatic tremor and reduced vibratory sense in lower limbs. On the SCA panel, *ATXN2* alleles had 22/38 repeats. She lost follow-up visits in 2015, at 60 years of age.

**3.6 Ataxia-Oculomotor Apraxia 2 (AOA2)**

Two cases had clinical suspicion of AOA2 that were later confirmed as carrying pathogenic variants in *SETX* (**Table 5** of the main manuscript).

With gait ataxia since age 14, case QC4063T was a man that started investigation at his 18 years of age, when he presented gait and limb ataxia, areflexia, oculomotor apraxia, high AFP (25 ng/mL) and axonal sensorimotor neuropathy. Without consanguineous parents nor recurrence in his sibship, Sanger sequencing of *SETX* detected a homozygous mutation. He walked independently until his 24 years of age when he started using bilateral support. In the last visit, with 29 years in 2024, his SARA was 21 and he was wheelchair bound.

Case YC8760Y was a woman that came to our attention at 20 years of age, complaining of gait ataxia followed by double vision and dysphagia since her 14 and 18 years of age. Her neurologic examination presented ataxic but independent gait, dysmetria, dystonic posture of right hand and foot, convergent strabismus, gazed-evoked nystagmus and absence of oculomotor apraxia. Her AFP was high (20 ng/mL). A cervical dystonic tremor started at 24 years of age. She obtained a court order for the state to perform Sanger sequencing of *SETX* at age 29 that confirmed the suspicion of AOA2 (**Table 5** of the main manuscript). SARA scores were 9.5 at 33 and 11.5 at 34 years of age, in her last visit in 2024.

**3.7 Ataxia-Oculomotor Apraxia 1 (AOA1)**

Case UAX78440 was a girl that started with gait ataxia at 3 years of age, beginning her investigation at 11 years, in 2009. The family denied consanguinity and reported that she presented intellectual disability and temper tantrums. Neurologic examination showed gait and limb ataxia, oculomotor apraxia, absent reflexes, and facial dyskinesias. AFP was normal and the clinical hypothesis was AOA1. She progressed to anarthria and wheelchair bound at age 15. A Sanger panel of AOA genes was obtained when she was 19 years of age, in 2017, and detected a biallelic mutation in *APTX* (**Table 5** of the main manuscript). In her last visit, in 2024, she was 26 years-old and her SARA scored 32 points.

**3.8 Sensory ataxic neuropathy, dysarthria, and ophthalmoparesis due to *POLG* (SANDO-*POLG*)**

Case IU6236F was a female patient with consanguineous parents whose initial clinical manifestations were gait ataxia and double vision at 19 years of age. She started evaluation at 21 years, when she presented gait and limb ataxia, nystagmus and absent ankle reflex. SARA scores were 5 at 24, 11 at 25, and 23 at 28 years of age. In 2015, with 28 years of age, an exome sequencing diagnosed a biallelic mutation in *POLG1*, compatible with SANDO-*POLG* (**Table 5** of the main manuscript). CoQ10 400mg was started and the next SARA scores were quite stable from 2016 to 2024: 24.5 at 29 yo, 22 at 30, 19.5 at 31, 21.5 at 32 and 34 yo, 24.5 at 35, and 25 points at 36 and 37 yo. Case IU6236F participated in a previous report from our study group [41] (Scott et al 2025).

**3.9 Joubert syndrome**

A 10 years-old girl started her investigation for presenting a neuropsychomotor developmental delay since 8 months of age plus gait deterioration since 3 years of age. Her neurological examination disclosed a cognitive deficiency, gait and limb ataxia, brisk reflexes, and oculomotor apraxia. Her MRI showed molar tooth sign and vermis cerebellar agenesis, findings that characterised the malformative Joubert syndrome. There was no definitive molecular diagnosis. As the patient was female, a rare X-linked inheritance pattern was considered unlikely, and the family received a diagnosis of Joubert syndrome with probable autosomal recessive inheritance.

**3.10 Spastic paraplegia 7 (SPG7)**

Case CI7789S was a female patient with gait ataxia since age 36 and with recurrence among sibs, described elsewhere [26] (Fussiger et al 2023). She started investigation at 42 years of age, in 2016, when she presented with gait and limb ataxia, dysarthria, nystagmus, spasticity of the lower extremities, and hyperreflexia. SPG panels were obtained for her and her affected sister when she was 46 years (in 2020) and detected two variants in both subjects that were considered likely pathogenic (PM3 strong criteria and recurrency) and diagnostic of SPG7 (**Table 5** of the main manuscript). The clinical picture was stable by 2024.

**3.11 Spastic paraplegia 11 (SPG11)**

Case PT1667H, already described [42] (Fussiger et al 2023), was a male patient that started with school difficulties, intentional tremor and gait ataxia at 12 years of age. Firstly seen at age 17 in 2011, he presented cognitive impairment, gait and limb ataxia, spastic paraparesis and pyramidal signs. His MRI revealed callosal atrophy and a periventricular leukodystrophy in 2011. In 2013, the “ears of the lynx”, characteristic of SPG11, was also seen. An SPG Sanger panel was obtained in 2018, by his 23 years of age, and detected two nonsense mutations at *SPG11* (**Table 5** of the main manuscript). His clinical picture was slightly deteriorated in his last visit, in 2024.

**3.12 Spastic paraplegia 76 (SPG76)**

Case EO6140R, described elsewhere [26] (Fussiger et al 2023), was a woman that started at 31 years of age with walking difficulties described as gait ataxia by her neurologist. She started her evaluation at 38 years, in 2011, when her neurologic examination showed a predominantly spastic gait, mild limb ataxia, dysarthria, brisk reflexes and Babinski sign. In 2020, a genome sequencing detected a biallelic mutation in *CAPN1* compatible with SPG76 (**Table 5** of the main manuscript). Her clinical picture was considered almost stable in follow-up visits until 2024.

**3.13 Short-chain enoyl-CoA hydratase (ECHS1) deficiency**

Case EB3490W was a female that presented neuropsychomotor developmental delay and walked at 3 years of age with gait ataxia. She started evaluation at 8 years of age, in 2019, when her examination disclosed a mild cognitive impairment, gait and limb ataxia, dysarthria, and nystagmus. An exome sequencing detected pathogenic variants in *ECHS1* (**Table 5** of the main manuscript), compatible with the diagnosis of ECHS1 deficiency. She received counseling about the risk of having metabolic acidosis crises and on agents/circumstances to avoid, such as mitochondrial toxins (valproic acid, prolonged propofol infusions) and a ketogenic diet. She also received instructions about the need to assess her acid-base status and blood lactate levels in case of infections or metabolic stressors. In the follow-up visits, bradykinesia, cervical dystonic tremor and mild spasticity in lower limbs were added to her ataxia syndrome. SARA scores were 10 and 12 and 2023 and 2024, at ages 12 and 13, respectively.

**3.14 Autosomal recessive spinocerebellar ataxia type 16 (SCAR 16)**

Case XD5260W was a female that started gait ataxia and investigation at 27 and 28 years of age, respectively. She presented with gait and limb ataxia, dysarthria, nystagmus, spasticity, and hyperreflexia in the lower limbs. Follow-up visits detected facial dyskinesia and dystonic movements in her neck and feet since her 33 years of age; with 34 years, her SARA was 20.5. An exome was obtained in 2022 by age 39 and detected variants in *STUB1*, diagnostic of SCAR16 (**Table 5** of the main manuscript). She was stable on her last visit, in 2024.

**3.15 Perrault syndrome**

Case EG5700Z was a female patient that started investigation in 2010, with 12 years of age, with a history of gait ataxia since her 10 years. Her examination disclosed gait and limb ataxia, nystagmus, and hyperreflexia in the lower limbs. A sensorineural hearing loss began abruptly at age 25. The other neurologic manifestations were stable during many years, with a SARA score of 11 in 2024, at age 26, when she obtained na exome sequencing that detected mutations in *HSD17B4* suggestive of Perrault syndrome (**Table 5** of the main manuscript). Using contraceptives and without symptoms of hypoestrogenism, her evaluation detected hyperprolactinemia.

**3.16 Developmental and epileptic encephalopathy-26 (DEE26)**

Case OV9196R was a male patient with neurodevelopmental delay since the age of 12 months, followed by petit mal spells. He started investigating at 6 years of age, when he presented cognitive impairment, gait and limb ataxia, dysarthria, spasticity and clonus in lower limbs. In 2023, with 13 years of age and with a stable clinical picture, an exome sequencing detected variants at *KCNB1* diagnostic of DEE26 (**Table 5** of the main manuscript**)**. The pathogenic variant detected was NM_004975.4(KCNB1):c.1184G>A. His parents were healthy; it was concluded that this dominant variant arose through a *de novo* mutation..

**3.17 Spinocerebellar Ataxia 27B (SCA 27B)**

Case RC1670Q was a male patient that started with gait ataxia and with investigation at ages 65 and 77, respectively. He reported on a 73 years-old brother with parkinsonian symptoms that was unable to come to evaluation. His complaints included a tendency to fall to either side that worsened in the last years, without dizziness or vertigo, followed by recent urinary incontinence, weakness in the hands and feet, dysphagia and double vision. His neurological examination showed gait and limb ataxia, dysarthria, nystagmus, hypermetric saccades, and reduced vibratory sense in lower limbs. The final diagnosis was established through a study that detected 9/338 GAA repeats at the *FGF14*-SCA27B repeat locus in intron 1 of *FGF14,* a finding that diagnosed SCA27B in 2024. In the same year, when the patient had 84 years of age, SARA score was 19. Treatment with 4-aminopyridine was discussed with the patient and his family.
